# Lung clearance index is elevated in young children with symptom‐controlled asthma

**DOI:** 10.1002/hsr2.58

**Published:** 2018-06-19

**Authors:** Christine Racette, Zihang Lu, Krzysztof Kowalik, Olivia Cheng, Glenda Bendiak, Reshma Amin, Aimee Dubeau, Renée Jensen, Susan Balkovec, Per Gustafsson, Felix Ratjen, Padmaja Subbarao

**Affiliations:** ^1^ Division of Respiratory Medicine, Department of Pediatrics Hospital for Sick Children and Research Institute Toronto Ontario Canada; ^2^ Dalla Lana School of Public Health University of Toronto Toronto Ontario Canada; ^3^ Department of Physiology University of Toronto Toronto Ontario Canada; ^4^ Department of Pediatrics University of Calgary Calgary Alberta Canada; ^5^ Department of Pediatrics Central Hospital Skövde Sweden

**Keywords:** asthma, lung clearance index, multiple breath washout, pediatric, preschool, pulmonary function testing, ventilation inhomogeneity

## Abstract

**Background:**

Pulmonary function testing has been recommended as an adjunct to symptom monitoring for assessment of asthma control. Lung clearance index (LCI) measures ventilation inhomogeneity and is thought to represent changes in the small airways. It has been proposed as a useful early marker of airway disease in asthmatic subjects, and determining it is feasible in preschool children. This study aims to assess whether LCI remains elevated in symptomatically controlled asthmatic children with a history of severe asthma, compared with healthy controls. A secondary aim was to determine whether the results were consistent across the preschool and school‐aged populations.

**Methods:**

Using a case‐control design, we compared 33 children with currently well‐controlled symptoms who had a history of severe asthma, to 45 healthy controls (age 3‐15 years) matched by age, height, and sex. We performed multiple breath washout tests using sulfur hexafluoride as a tracer gas, to determine their LCI and S_cond_ values.

**Results:**

In the overall study, LCI z‐score values were on average 0.86 units (95% confidence interval: 0.24‐1.47, *P* = 0.01, t‐test) higher in children with a history of severe asthma with current well‐controlled symptoms compared with healthy controls. In addition, within the subgroup of preschool children (age ≤ 6), the asthmatic had significantly higher LCI z‐score values than their healthy controls peers (mean (SD), 0.57 (2.18) vs −1.10 (1.00), *P* = 0.03, t‐test). Twenty‐seven percent (27%; 9/33) of subjects had an LCI value greater than the upper limit of our healthy controls despite being symptom controlled. Amongst preschool children, 5 (42%; 5/12) of the asthmatic children had abnormal LCI at the individual level.

**Conclusions:**

LCI is elevated in children with asthma, which may be driven by differences in the preschool population. LCI may be useful in defining preschool asthma endotypes with persistent ventilation inhomogeneity despite symptomatic control.

**Abbreviations**
FeNO: Fractional exhaled nitric oxide
FEV_1_: Forced expiratory flow in 1 second
FRC: Functional residual capacity
FVC: Forced vital capacity
GLI: Global Lung Initiative
LABA: Long‐acting beta‐agonist
LCI: Lung clearance index
MBW: Multiple breath washout
N_2_: Nitrogen gas
SF_6_: Sulfur hexafluoride
ULN: upper limit of normal

## 1 INTRODUCTION

Asthma is characterized by airway inflammation and airway hyper‐reactivity, leading to variable airflow obstruction and associated respiratory symptoms.^1^ Clinical evaluation and spirometry are the current standards for the diagnosis and monitoring of asthma.^1^ Many longitudinal cohorts report decrements in spirometry in children with asthma (compared with healthy children) that appear by school‐age and persist through to adulthood,^2^, ^3^, ^4^ yet, in practice, spirometry is often within the normal range in pediatric asthma patients and is challenging to perform in preschool asthmatics, where burden of disease is highest.^5^

Although asthma is a disease of both central and peripheral airways,^6^, ^7^, ^8^ FEV_1_, a spirometry‐based test normally required for the objective assessment of asthma severity, measures mainly central airways obstruction. The small airways have been called the “quiet zone”,^8^, ^9^ alluding to the challenges in its evaluation using conventional spirometric maneuvers. Therefore, interest in the development of technologies tailored to recognize early peripheral airway changes in asthma, feasible for all ages, has been a priority.

Lung clearance index (LCI), attained during the multiple breath washout (MBW) test, measures ventilation inhomogeneity. Because MBW depends on tidal breathing measures, it is feasible to measure in very young children. It has been suggested in the literature that LCI represents changes in the peripheral airways.^10^ Airway biopsy studies suggest that inflammation persists in the small peripheral airways despite therapy, and in the presence of normal range spirometry.^11^ Very little data exists in the literature about LCI in children with asthma and whether it may offer insight into endotypes of pediatric asthma. A study by Sonnappa et al^12^ suggested that MBW indices were elevated in children with multi‐trigger wheeze compared with healthy controls. This study included symptomatic children and did not include an assessment of symptom control. Another study in older children also documented elevated LCI values compared with healthy controls, but again, no assessment of symptom control was made.^13^ Magnetic Resonance Imaging data suggest persistent ventilation defects are present in children with preschool asthma, the size of which correlates with disease severity.^14^ It is not clear if LCI, which provides a global estimate of lung ventilation heterogeneity, may provide insight into lung physiologic changes in asthmatic children who despite a history of severe exacerbations, are currently under symptomatic control.

The aim of this study was to determine whether LCI remained elevated in a cross‐sectional cohort of asthmatic children with a history of severe exacerbations who currently had well‐controlled symptoms of asthma compared with healthy controls. A secondary aim was to explore age‐related differences in LCI amongst the asthmatic children. We hypothesized that LCI would be higher in asthmatic children compared with healthy controls despite their symptoms being well controlled.

## 2 MATERIALS AND METHODS

### 2.1 Study design and participants

We designed a case‐control study in which children with asthma (cases) were compared with healthy children (controls).

Children (3‐18 years old) with asthma were recruited from the severe Asthma outpatient clinics at the Hospital for Sick Children between December 2009 and May 2015. All children who were approached for this study had specialist physician‐confirmed asthma that included the following: a history of physician‐documented wheeze on >2 occasions, and either a clinical response to a bronchodilator or a bronchodilator response of greater than 12% change in FEV_1_. All children had a history of at least 1 hospitalization or >2 courses of oral corticosteroids within the past year for uncontrolled asthma symptoms or a history of uncontrolled asthma symptoms despite adequate treatment (Step 3‐5 NHLBI treatment). For inclusion into this study, children with physician‐diagnosed asthma had to have evidence of current symptom control, defined as: use of a short‐acting bronchodilator <3 times per week; daytime asthma symptoms <3 times per week, and nocturnal awakenings due to their asthma ≤1 per week. Children ≥6 years of age able to perform spirometry had to have an FEV_1_ in the normal range (> 80% after race‐correction based on the reference equations from Global Lung Initiative (GLI)^15^). Exclusion criteria included prematurity or low birth weight, a history of congenital heart disease, neuromuscular disorder or bone disease, a history of chronic lung disease other than asthma, respiratory infection or change in medication within the past 3 weeks, and history of smoking. Symptoms and medical history were obtained by questionnaire and chart review.

Healthy children were recruited from the general population to serve as the control group. Exclusion criteria included prematurity or low birth weight, a history of congenital heart disease, neuromuscular disorder or bone disease, history of chronic productive cough or recurrent wheezing or shortness of breath within the last 12 months, a history of any chronic lung disease including a diagnosis of asthma or reactive airway disease, any previous hospital admission for a respiratory condition, and history of smoking.

This study received approval from the Hospital for Sick Children research ethics board (REB # 1000013927), and consent was obtained from all parents or participants, where applicable.

### 2.2 Pulmonary function tests

Pulmonary function was performed in a standardized order: MBW followed by spirometry. In children able to perform acceptable and reproducible spirometry testing, as per our clinical protocol, bronchodilator (Salbutamol 400 μg) was administered using a holding chamber. Post‐bronchodilator spirometry and MBW testing were repeated 15 minutes after bronchodilator administration.

#### 2.2.1 Multiple breath washout (MBW)

MBW tests were performed using a facemask (Silkomed, Benson Medial Industries, ON) sealed with therapeutic putty (Air Putty, Sammons Preston Canada Inc, ON) for the preschool‐aged children, or a mouthpiece (VacuMed, Ventura, CA, USA) for the school‐aged children, attached to a heated pneumotachometer (Hans Rudolph, KS, United States). A test gas containing 4% sulfur hexafluoride (SF_6_) was used to measure LCI, using an AMIS 2000 mass spectrometer (Innovision A/S, Odense, Denmark). Washout testing was completed when the test gas concentration fell below 1/40^th^ of the initial concentration. All MBW trials were recorded and analyzed using a custom data acquisition and offline analysis program (TestPoint, Measurement Computing, Norton, MA, USA). Quality control was assessed post‐testing as per the ATS/ERS statement^16^ and included a stable wash‐in with little fluctuation of the SF_6_ signal, a clean disconnect below 0.1% SF_6_, a clean, regular, and representative functional residual capacity (FRC) breath (before disconnect), and finally, a stable and steady breathing pattern. Pre‐gas sampling point and post‐gas sampling point dead space was set to 0 and 0.0154 L, respectively. LCI, calculated as the number of lung turnovers required to reach a concentration in the tracer gas equal to 1/40^th^ of the starting concentration, is derived from the TestPoint software, as are S_cond_ values, as previously described.^17^Quality control for the breath‐by‐breath analysis was performed first, before an S_cond_ value was generated. As per previously described,^18^ we excluded any breaths in each trial with >25% deviation from the mean tidal volume and any trials that had <66% of the breaths remaining after exclusion.

#### 2.2.2 Spirometry

Spirometry was performed using the Vmax Encore system (CareFusion, San Diego, CA, USA) and reported as per the ATS/ERS guidelines.^19^, ^20^ Spirometry z‐scores and percent predicted values were calculated using the GLI equations.^15^ Participants were grouped into self‐reported ethnic groups, and parameters were race‐corrected, as per GLI equations.

### 2.3 Atopy

Aeroallergen sensitization was defined as a wheal size ≥2 mm larger than the saline control, to a standard panel of 14 aeroallergens. These aeroallergens included house dust mites (*Dermatophagoides farine* and *pteronyssinus*), cockroach, cat, dog, mouse, horse, feathers, tree mix, grass mix, ragweed, alternaria, hormodendrum, aspergillus mix. Eczema and allergic rhinitis history was based on parental report of a past diagnosis or typical symptoms. Atopic status was defined as any of the following: aeroallergen sensitization, a history of eczema, or atopic rhinitis. Atopy sensitization in controls was not assessed.

### 2.4 Statistical analysis

Height, weight, and body mass index (BMI) centiles were calculated using WHO growth charts (WHO 2006).^21^ Healthy controls and asthma children were matched by age, height, and sex. Baseline population characteristics were presented as median (interquartile range) or frequency (percentage), where appropriate. Comparisons between healthy and asthma groups for baseline population characteristics were performed using Student t‐test or Mann‐Whitney U test, where appropriate, for continuous variables, and chi‐square test, for categorical variables.

The primary statistical analysis was to determine whether LCI remained elevated in asthmatic children with a history of severe exacerbations who currently had well‐controlled symptoms of asthma, compared with healthy controls. Given our sample size of 45 healthy and 30 asthmatic children, we were able to detect a 0.7 z‐scores difference and 0.8 z‐scores difference between the 2 groups, with 80% and 90% power, respectively, at 0.05 significance level. Lung function parameters were presented as mean (standard deviation, SD) and analyzed both as raw scales and z‐scores, calculated using the published reference equations for MBW^22^ and spirometry.^15^ To further investigate MBW (eg, LCI) and spirometry parameters (eg, FEV_1_) in children with asthma compared with healthy controls, linear regression models were used, and difference and its associated 95% confidence interval (CI) between the 2 groups were estimated. The analyses were adjusted for age and height values when raw scale values were used, whereas no adjustment were performed when z‐score or percent predicted values were used.

To investigate the secondary aim of age‐related differences in LCI, we included an interaction term between age and group in the linear regression models to test its significance. In addition, we performed a subgroup analysis by dividing the children into preschool‐age children (age ≤ 6) and school‐age children (age > 6). Comparison between asthmatic and healthy controls within each age group was performed using 2‐sample t test or Mann‐Whitney U test, where appropriate. Correlations between lung function parameters and clinical factors were assessed using a Spearman's correlation. According to ATS/ERS recommendation,^23^ the lower limit of normal and upper limit of normal (ULN) of lung function parameters were defined as 5^th^ and 95^th^ percentile of our healthy controls, respectively. Comparisons of lung function parameters between pre and post BD were performed using paired t‐test. Statistical analyses were performed using SAS version 9.4 (SAS Statistical Software, Cary, NC, USA). Statistical tests were 2 sided, and significance level was set at *P* < 0.05.

## 3 RESULTS

### 3.1 Study participants

The current study included 33 asthmatic and 45 healthy controls children matched for age, height, and sex. Demographic comparisons between the 2 groups are presented for the overall age (Table 1), and by preschool‐age and school‐age (Table 2). Asthma patients were predominantly atopic (30/33; 91%) and had moderate to severe asthma (23/33; 70%), as defined using the NHLBI classification of asthma severity^1^ (Table 3). In the year preceding their involvement in the study, 42% (14/33) used oral steroids, 42% (14/33) visited the emergency room for asthma, and 21% (7/33) were hospitalized for asthma.

**Table 1:** Demographic comparisons between asthmatic and healthy children

aVariables	Healthy *N* = 45	Asthma *N* = 33	* *P* Value
Height (cm)	133.00 (116.50, 145.50)	136.50 (106.00, 151.50)	0.77
Weight (kg)	30.00 (21.70, 38.80)	37.40 (17.80, 43.60)	0.97
BMI	17.01 (15.98, 18.61)	16.91 (15.87, 19.38)	0.86
BMI z‐score	0.36 (−0.06, 1.01)	0.47 (0.06, 0.94)	0.88
Height z‐score	0.40 (−0.37, 1.07)	−0.04 (−0.36, 0.54)	0.1
Age (year)	8.01 (5.90, 11.09)	9.96 (5.09, 11.84)	0.95
Male, *N*(%)	29 (64%)	25 (76%)	0.33
Caucasian, *N*(%)	35 (78%)	19 (58%)	0.08

a Variables are presented as median (interquartile range) unless otherwise specified.

* *P* values were calculated using Student t‐test or Mann‐Whitney U test, where appropriate, for continuous variables, and chi‐square test for categorical variables.

**Table 2:** Demographic comparisons between asthmatic and healthy children by preschool‐age and school‐age

	Age ≤ 6			Age > 6		
aVariables	Asthmatic *N* = 12	Healthy *N* = 13	* *P* Value	Asthmatic *N* = 21	Healthy *N* = 32	* *P* Value
Height (cm)	104.50 (101.50, 108.00)	112.50 (104.00, 116.50)	0.05	149.00 (137.00, 154.00)	141.75 (130.50, 153.75)	0.29
Weight (kg)	17.10 (15.90, 18.40)	20.30 (17.00, 21.70)	0.02	42.50 (38.70, 49.00)	35.60 (29.05, 46.50)	0.17
BMI	15.80 (15.02, 16.12)	16.02 (15.21, 16.41)	0.26	19.00 (16.98, 21.80)	17.49 (16.19, 19.63)	0.17
BMI z‐score	0.38 (−0.17, 0.58)	0.52 (−0.06, 0.86)	0.34	0.73 (0.28, 1.64)	0.27 (−0.10, 1.04)	0.5
Height z‐score	−0.09 (−1.06, 0.29)	0.69 (−0.40, 1.15)	0.08	0.28 (−0.34, 0.60)	0.35 (−0.35, 1.05)	0.51
Age (year)	4.40 (3.90, 5.18)	4.88 (4.19, 5.78)	0.38	11.43 (10.03, 12.38)	10.40 (7.87, 12.07)	0.18
Male, *N*(%)	7 (58%)	10 (77%)	0.41	18 (86%)	19 (59%)	0.07
Caucasian, *N*(%)	8 (67%)	11 (85%)	0.38	11 (52%)	24 (75%)	0.14

a Variables are presented as median (interquartile range) unless otherwise specified.

* *P* values were calculated using Student *t*‐test or Mann‐Whitney U test, where appropriate, for continuous variables, and chi‐square test for categorical variables.

**Table 3:** Clinical characteristics and treatment care of children with clinically stable asthma at time of testing

Clinical Characteristics	Overall *N* = 33	Age ≤ 6 *N* = 12	Age > 6 *N* = 21
**Asthma risk factors**			
Atopy	30 (91%)	12 (100%)	18 (85.7%)
Eczema	20 (61%)	7 (58.3%)	13 (61.9%)
Allergic rhinitis	23 (70%)	6 (50%)	17 (81%)
Sensitivity to ≥1 aeroallergens	24 (73%)	8 (66.7%)	16 (76.2%)
First degree relative with asthma	21 (64%)	6 (50%)	15 (71.4%)
First degree relative with eczema and/or rhinitis	23 (70%)	8 (66.7%)	15 (71.4%)
**Treatment and health care**			
**Regular controller therapy**	31 (94%)	11 (92%)	20 (95%)
Inhaled corticosteroids only	14 (42%)	9 (75%)	5 (24%)
Inhaled corticosteroids + LABA only	4 (12%)	0	4 (19%)
Montelukast only	2 (6%)	0	2 (10%)
Multiple controllers	11 (33%)	2(17%)	9(43%)
**NHLBI treatment step**			
Steps 1‐2 (mild)	10 (30%)	4(33.3%)	6 (28.6%)
Steps 3‐5 (moderate‐severe)	23 (70%)	8 (66.7%)	15 (71.4%)
Hospitalized in the last 12 months	7 (21%)	3 (25%)	4 (19.1%)
Visited ER for asthma in the last 12 months	14 (42%)	6 (50%)	8 (38.1%)
Received oral corticosteroids in the last 12 months	14 (42%)	6 (50%)	8 (38.1%)

**Figure 1:**
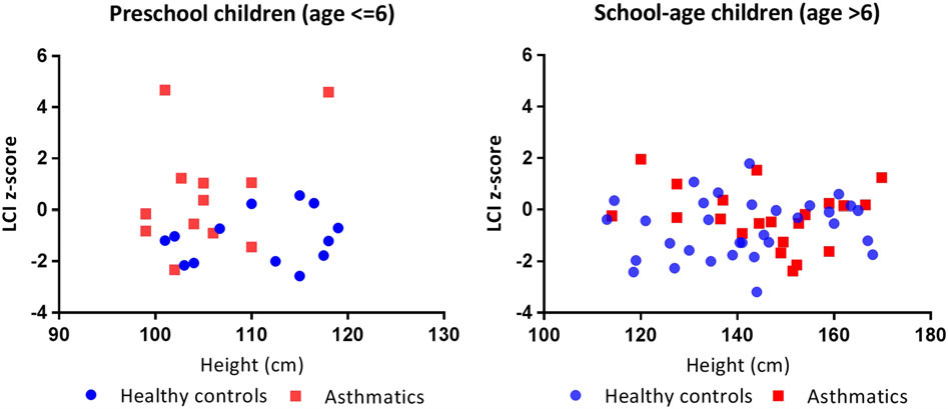
LCI z‐score plotted against height by age groups. Left panel: LCI z‐score vs height for preschool children (age ≤ 6). Right panel: LCI z‐score vs height for school‐age children (age > 6)

**Figure 2:**
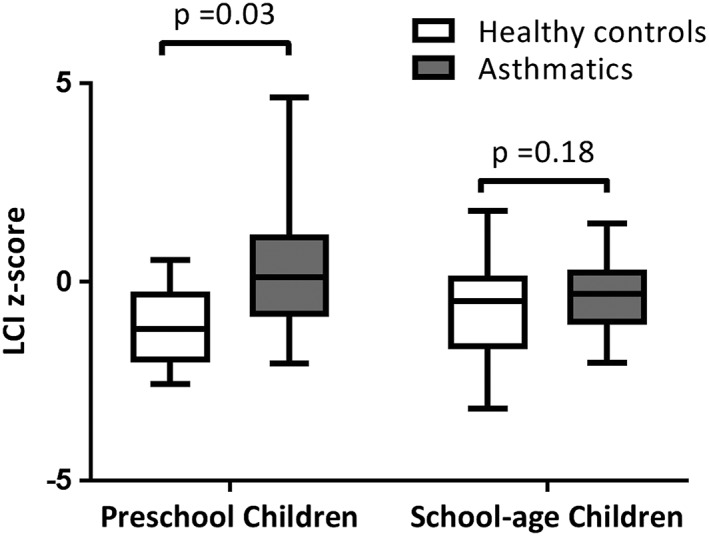
Comparison of LCI z‐score between asthmatics (*N* = 12) and healthy controls (*N* = 13) in preschool children (age ≤ 6) and between asthmatics (*N* = 21) and healthy controls (*N* = 32) in school‐age children (age > 6). The lines and whiskers in the boxplot denote median and ranges, respectively. *P* values were calculated using *t*‐test

Of the children included in the current study, 26 (79%; 26/33) asthmatic and 38 (84%; 38/45) healthy children had repeatable and acceptable FEV_1_ measures, whereas 25 (76%; 25/33) asthmatic and 27 (60%; 27/45) healthy children had repeatable and acceptable FEV_1_/FVC measures. This population is ethnically diverse and, therefore spirometry data were standardized through calculating the z‐scores and percent predicted values. FEV_1_% predicted was comparable between the asthmatic children and healthy controls (mean (standard deviation, SD): 97 (12.5) vs 100.1 (11.3), *P* = 0.31 t‐test) (Table 4). The asthmatic children had a statistically significantly lower FEV_1_/FVC (% predicted) compared with the healthy control group (mean (SD), 92.5 (8.1) vs 97.5 (8.4), *P* = 0.05 t‐test). At the individual level, 1 child with asthma (4%; 1/25) had an FEV_1_/FVC (% predicted) value that was lower than the lower limit of normal (<76.68, ie, 5^th^ percentile of our healthy controls). For MBW measures, we found that children with asthma had a mean (95% confidence interval) of 0.86 (95% CI: 0.24‐1.47) units higher LCI z‐score compared with healthy controls (mean (SD), 0.03 (1.62) vs ‐0.83 (1.10), *P* = 0.01, t‐test) in the overall age group (Table 4). We repeated the analysis by removing 2 subjects with extreme LCI values, and our results remained robust, that is LCI is still elevated in asthmatic children compared with healthy controls in the overall population (*P* = 0.03, t‐test). In addition, we found that the age group (preschool vs school‐age) had a significant interaction effect on LCI z‐score (*P* = 0.04, t‐test of coefficient from linear regression), indicating that there was an age‐related different in LCI z‐score between the 2 groups. This was confirmed with the analysis within subgroups of preschool‐age children (age ≤ 6) and school‐age children (age > 6) (Figure 1). The preschool asthmatic children had significantly higher LCI z‐score values than that in their healthy controls peers (mean (SD), 0.57 (2.18) vs −1.10 (1.00), *P* = 0.03, t‐test). Although the LCI z‐score remained higher in asthmatic children (mean (SD), −0.25 (1.18) vs −1.10 (1.00)), this difference became non‐significant (*P* = 0.07, t‐test) after removing the 2 subjects with extreme LCI values. This may due to the limited sample size and lack of power within this restricted age range. For school‐age children, the difference of LCI z‐score between the asthmatic and healthy controls children was not statistically significant (mean (SD), −0.28 (1.15) vs −0.72 (1.13), *P* = 0.18, t‐test) (Table 5 and Figure 2). Likewise, this non‐significance may due to the limited sample size and lack of power within this subgroup. In the overall population, the FRC z‐score did not differ between the asthmatic and healthy group (mean (SD), −0.37 (1.02) vs −0.54 (1.20), *P* = 0.51, t‐test). In addition, the FRC z‐score was similar between the 2 groups in preschool‐age (mean (SD), −0.31 (0.96) vs 0.28 (1.03), *P* = 0.16, t‐test) as well as in school‐age (mean (SD), −0.41 (1.07) vs −0.88 (1.11), *P* = 0.14, t‐test). The 2 subjects who had extreme outliers for LCI z‐score had FRC z‐score of −1.73 and −1.94 that were lower than average for the asthmatic population. S_cond_ values were significantly higher in asthmatic children compared with the healthy controls (mean (SD), 0.04 (0.03) vs 0.02 (0.02), *P* = 0.02, t‐test) (Table 4). This difference remained significant in preschool‐age children (mean (SD), 0.05 (0.03) vs 0.03 (0.02), *P* = 0.05, t‐test); however, it was nonsignificant in school‐aged children (mean (SD), 0.02 (0.02) vs 0.02 (0.01), *P* = 0.2, t‐test) (Table 5).

**Table 4:** Comparison of lung function parameters between asthmatics and healthy controls across all age

aVariables	*N*	Asthmatic	*N*	Healthy	Differences (95%CI) Between Asthmatic and Healthy Children	* *P* Value
LCI	33	6.71 (1.11)	45	6.18 (0.51)	0.53 (0.16, 0.91)	0.01
LCI z‐score	33	0.03 (1.62)	45	−0.83 (1.10)	0.86 (0.24, 1.47)	0.01
FRC	33	1.16 (0.66)	45	1.10 (0.54)	0.06 (−0.07, 0.19)	0.34
FRC z‐score	33	−0.37 (1.02)	45	−0.54 (1.20)	0.17 (−0.34, 0.68)	0.51
S_cond_	25	0.04 (0.03)	33	0.02 (0.02)	0.02 (0.003, 0.03)	0.02
FEV_1_ (% predicted)	26	97.0 (12.5)	38	100.1 (11.3)	−4.1 (−9.9, 1.7)	0.16
FEV_1_ z‐score	26	−0.23 (1.02)	38	0.02 (0.93)	−0.25 (−0.74, 0.24)	0.31
FEV_1_/FVC (% predicted)	25	92.5 (8.1)	27	97.5 (8.4)	−4.6 (−9.3, 0.09)	0.05
FEV_1_/FVC z‐score	25	−0.99 (1.11)	27	−0.25 (1.14)	−0.74 (−1.36, −0.11)	0.02

a Variables are presented as mean (SD).

* *P* values were calculated from *t* tests of coefficients from linear regressions. The analyses were adjusted for age and height values when raw scale values were used, whereas no adjustment were performed when z‐score or percent predicted values were used.

**Table 5:** Comparison of lung function parameters between asthmatics and healthy controls by age groups

	Age ≤ 6					Age > 6				
aVariables	*N*	Asthmatic	*N*	Healthy	* *P* Value	*N*	Asthmatic	*N*	Healthy	* *P* Value
LCI	12	7.24 (1.56)	13	6.14 (0.46)	0.02	21	6.41 (0.61)	32	6.20 (0.54)	0.21
LCI z‐score	12	0.57 (2.18)	13	−1.10 (1.00)	0.03	21	−0.28 (1.15)	32	−0.72 (1.13)	0.18
FRC	12	0.54 (0.10)	13	0.68 (0.12)	<0.01	21	1.51 (0.59)	32	1.27 (0.55)	0.16
FRC z‐score	12	−0.31 (0.96)	13	0.28 (1.03)	0.16	21	−0.41 (1.07)	32	−0.88 (1.11)	0.14
S_cond_	10	0.05 (0.03)	12	0.03 (0.02)	0.05	15	0.02 (0.02)	21	0.02 (0.01)	0.2
FEV_1_ (% predicted)	5	93.0 (18.0)	7	92.2 (12.6)	0.93	21	97.95 (11.3)	31	101.9 (10.4)	0.15
FEV_1_ z‐score	5	−0.5 (1.37)	7	−0.59 (0.95)	0.94	21	−0.15 (0.95)	31	0.16 (0.88)	0.16
FEV_1_/FVC (% predicted)	4	101.5 (4.7)	4	98.4 (15.2)	0.77	21	90.8(7.5)	23	97.3 (7.3)	0.01
FEV_1_/FVC z‐score	4	0.27 (0.84)	4	0.21 (1.85)	0.96	21	−1.23 (1.00)	23	−0.33 (1.01)	0.01

a Variables are presented as mean (SD).

* *P* values were calculated using 2‐sample Student *t*‐test or Mann‐Whitney U test, where appropriate.

At the individual level, 9 children with asthma (27%; 9/33) had an LCI value that fell above the ULN of our healthy control population (> 6.94, ie, 95^th^ percentile of our healthy controls) despite being under symptomatic control. Amongst preschool children, 5 (42%; 5/12) of the asthmatic children had abnormal LCI at the individual level. None of the clinical factors (past hospitalizations, use of oral steroids or emergency visits, type of controller therapy, treatment level or dosage) were significantly associated with abnormal LCI. There was also no correlation between any of the spirometry parameters and LCI.

In the asthmatic group, the mean (SD) changes between post and pre bronchodilator in LCI raw scale and z‐score were 0.05 (0.58) and 0.02 (1.16), respectively, neither of which was significant (Table 6). Finally, although post‐BD FEV_1_ (% predicted) was statistically significantly higher compared with pre‐BD, the change of 3.9 (2), *P* < 0.001 (paired t‐test), which was less than the cut‐off 12% as recommended by ATS/ERS,^24^ was not considered clinically significant.

**Table 6:** Comparison of lung function values between pre and post BD in asthmatic group

aVariables	*N*	Pre BD	Post BD	Post‐Pre BD	* *P* Values
LCI	21	6.64 (1.02)	6.69 (1.23)	0.05 (0.58)	0.68
LCI z‐score	21	−0.06 (1.50)	−0.04 (1.80)	0.02 (1.16)	0.93
FRC	21	1.06 (0.49)	1.02 (0.46)	−0.04 (0.10)	0.12
FRC z‐score	21	−0.68 (0.97)	−0.83 (1.05)	−0.15 (0.58)	0.26
S_cond_	15	0.04 (0.03)	0.03 (0.03)	−0.01 (0.04)	0.35
FEV_1_ (% predicted)	16	98.5 (12.7)	102.4 (12.9)	3.9 (5.3)	0.01
FEV_1_ z‐score	16	−0.12 (1.06)	0.21 (1.10)	0.33 (0.45)	0.01
FVC (% predicted)	14	109.2 (17.0)	107.77 (17.0)	−1.4 (5.2)	0.33
FVC z‐score	14	0.77 (1.43)	0.65 (1.43)	−0.12 (0.44)	0.33
FEV_1_/FVC (% predicted)	14	90.6 (8.4)	94.5 (8.7)	3.9 (2.0)	<.0001
FEV_1_/FVC z‐score	14	−1.27 (1.13)	−0.73 (1.22)	0.54 (0.28)	<.0001

a Variables are presented as mean (SD).

* *P* values were calculated using paired t‐test.

## 4 DISCUSSION

We report that LCI values remain elevated in asthmatic children with a history of severe exacerbations deemed to be well‐controlled at the time of their outpatient assessment, compared with healthy controls matched by age, height, and sex. This difference in LCI may be driven by children less than 6 years of age, an age group in which symptom and lung function assessment can be challenging.^25^, ^26^, ^27^, ^28^, ^29^ Apart from age, our analyses did not identify any other factor predictive of an abnormal LCI. Notably, none of the clinical factors assessed, atopic status, FEV_1_ or FEV_1_/FVC ratio, were predictive of an abnormal LCI value. Taken together, these findings suggest that LCI may be useful in phenotyping lung function abnormalities in preschool children with asthma who are deemed to be well‐controlled by clinical features.

Similar to our study, Zwitserloot et al^30^ and Macleod et al^31^ measured LCI using SF_6_ washout. Zwitserloot et al^30^ found that a group of asthmatic children, described as clinically stable and treated with low doses of inhaled corticosteroids, had a significantly higher LCI than healthy controls. Similar to their study, we also found no correlation between FEV_1_ and LCI. A study by Macleod et al^31^ found a significantly higher LCI among well‐controlled children with asthma compared with healthy controls, which was not associated with an evidence of persisting airways inflammation based on FeNO measurements. Our study shows similar results for mean LCI among a younger, more atopic group. Keen et al^32^ also reported a significantly higher LCI among school‐aged children with allergic asthma compared with healthy controls, as well as an association between ventilation inhomogeneity and airway hyper‐responsiveness. The difference in LCI observed in the older population studied by Keen et al may be explained by the influence of children with uncontrolled asthma at the time of testing. Even though the asthma group showed a significantly higher LCI than controls in all of these studies, including ours, their mean LCI remained within the normal range, and only a small fraction had a markedly elevated LCI, suggesting a limited role of LCI in monitoring asthma control. By contrast, we noted that almost half of our preschool asthmatic population (42%; 5/12) had an LCI value above the ULN (> 6.94, ie, 95^th^ percentile of our healthy control range). Our population was recruited from a specialty tertiary care asthma clinic and may represent a more severe phenotype. Of note, while most (>90%) of the asthmatic children were on regular controller therapy, 9/12 (75%) preschool asthmatic children were on monotherapy whereas only 2/12 (17%) were on multiple controllers (Table 3). The use of additional therapy in the older age group could account for better control and, therefore, lower LCI values. In addition, LCI is calculated as the ratio of cumulative expired volume divided by FRC; therefore, small decreases in FRC would be magnified in the overall LCI calculation. Although the differences in FRC noted between the asthmatic and healthy children were quite similar in the overall population, amongst preschool children, the FRC measured by MBW was lower numerically in asthmatic children compared with healthy controls. This may have accounted for the elevated LCI values in this age group. We did not measure the FRC by plethysmography, only by MBW, and the decreased FRC may have been due to partial atelectasis. This hypothesis is supported in the literature in this age group by MRI imaging data showing ventilation defects.^33^

Comparable to previous studies,^30^, ^31^ we did not detect a bronchodilator response in LCI in our dataset. The absence of a bronchodilator response for children ≥6 years who performed post‐bronchodilator spirometry was part of our definition of clinically stable asthma; therefore, it was not surprising that no change was observed in LCI for our participants as well. However, half of the asthmatic participants with a high LCI were < 6 years old and were unable to perform a post‐bronchodilator spirometry measurement. The bronchodilator response in LCI was not repeated in those <6 years old and, therefore, may have underestimated the responsiveness of LCI to bronchodilator. We did not plan the study to assess the responsiveness of LCI but rather to determine if it was normal when children were well controlled symptomatically. Future studies to assess bronchodilator responsiveness of LCI should be planned prospectively in preschool children.

More recently, newer techniques have used hyperpolarized gases to assess ventilation defects, which may correspond to LCI assessment of ventilation inhomogeneity. These studies confirm that fixed ventilation defects are present in adult asthmatics^34^ despite having normal spirometry^35^ and may be refractory to therapy.^36^ Pediatric imaging studies confirm that fixed ventilation imaging defects are present in children with asthma^14^ and do not correlate with spirometric outcomes.^37^ Altes et al^14^ found that 77% of the children with asthma, regardless of age, had a ventilation defect which was typically in the lung periphery and, thus, perhaps, not easily assessed using spirometry. They also reported a correlation between the percentage of lung affected by a ventilation defect and worsening asthma severity, as defined by higher daily controller medications and prednisone use.^14^ Of note, these defects may represent areas of poorer ventilation and not necessarily completely non‐ventilated areas. Newer modalities of ventilation imaging studies such as multiple breath MRI imaging have the ability differentiate regions with slower ventilation and delayed filling compared with completely non‐ventilated areas. Only slowly filling ventilation defects contribute to an elevated LCI, whereas completely non‐ventilated areas may not contribute to gas mixing and, therefore, theoretically, would not increase LCI values. Narrowing of subsegmental small airways leading to poorer ventilation is consistent with asthma histopathology data showing active inflammation in the small airways despite normal pulmonary function.^38^ Functional measures of ventilation inhomogeneity were not included in this study, but it is tempting to speculate that the elevated LCI noted in our study may represent these persistent ventilation defects and may help to identify children with a more severe asthma phenotype. Longitudinal studies combining functional and imaging measures of ventilation inhomogeneity are needed to explore these findings.

Our study had several limitations. Firstly, we were limited in our ability to phenotype our preschool children with asthma using objective measures, because at the time of the study, our clinical laboratory had not instituted preschool spirometry. Half of all asthmatic children with abnormal LCI values were in the preschool range; therefore, interpretation of the relationship with spirometry is limited. Secondly, among the preschool children, the asthma subjects were shorter and weighed less compared with healthy controls (Table 2) which may be a reflection of their underlying disease. It is known that height is related to LCI values and, therefore, is accounted for in our analysis. Thirdly, our sample size is limited which may result in lack of power in our subgroup analysis. Larger studies in asthmatic and healthy children are needed to confirm our observations. Fourthly, our clinical laboratory did not standardly assess our asthmatics with markers of inflammation such as FeNO; thus, we are not able to relate LCI findings with markers of airway inflammation. Fifthly, our entry criteria of symptom control were based on standardized history but predate the development of the preschool standardized symptom assessment scores such as the TRACK score^39^; thus, symptom control may have been overestimated. Symptom assessment is known to be challenging in preschool children and the lack of a standardized symptom score limits our ability to comment on the relative utility of objective measures and clinical assessment in young children with asthma. Further work must be done to understand the implications of abnormal LCI values, including imaging and longitudinal studies of changes in LCI along with standardized symptom assessment. Future studies should focus on the preschool population to compare other viable standardized preschool asthma assessment tools to MBW measurement and assessments of disease severity. The final limitation pertains to the use of SF_6_ as a tracer gas for MBW. This technique is being replaced by nitrogen washout, and comparison of results from studies using a different gas must be interpreted with caution.

In summary, we showed that LCI is elevated in children with asthma despite physician‐assessed symptom control. This difference may be driven by the preschool population. The implications of an abnormal LCI require further investigation, including imaging and assessment of inflammatory phenotype. MBW testing may prove to be a useful monitoring tool in the assessment of asthma in young children. Future studies should assess the utility of different preschool lung function tests such as MBW and spirometry in assessing disease severity in preschool children with asthma.

## FUNDING

Don and Debbie Morrison and SickKids Foundation.

## CONFLICT OF INTEREST

None declared.

## AUTHOR CONTRIBUTIONS

Conceptualization: PS, CR, ZL

Investigation: PS, CR, ZL

Data Collection: PS, CR, ZL, KK, OC, GB, RA, AD, RJ, SB, PG

Formal analysis & Software: ZL

Interpretation of data: PS, CR, ZL, KK, OC, GB, RA, AD, RJ, SB, PG, FR

Writing—original draft: PS, CR, ZL

Writing—review and editing: PS, CR, ZL, KK, OC, GB, RA, AD, RJ, SB, PG, FR

All authors approved the final version of the manuscript.
